# Adverse effects of temperature on perinatal and pregnancy outcomes: methodological challenges and knowledge gaps

**DOI:** 10.3389/fpubh.2023.1185836

**Published:** 2023-11-01

**Authors:** Maitry Mehta, Rupa Basu, Rakesh Ghosh

**Affiliations:** ^1^Sawyer Business School, Suffolk University, Boston, MA, United States; ^2^California Environmental Protection Agency, Office of Environmental Health Hazard Assessment, Oakland, CA, United States; ^3^Institute for Health and Aging, University of California, San Francisco, San Francisco, CA, United States

**Keywords:** temperature, birth outcomes, pregnancy outcomes, heat, climate change

## Abstract

Evidence linking temperature with adverse perinatal and pregnancy outcomes is emerging. We searched for literature published until 30 January 2023 in PubMed, Web of Science, and reference lists of articles focusing on the outcomes that were most studied like preterm birth, low birth weight, stillbirth, and hypertensive disorders of pregnancy. A review of the literature reveals important gaps in knowledge and several methodological challenges. One important gap is the lack of knowledge of how core body temperature modulates under extreme ambient temperature exposure during pregnancy. We do not know the magnitude of non-modulation of body temperature during pregnancy that is clinically significant, i.e., when the body starts triggering physiologic counterbalances. Furthermore, few studies are conducted in places where extreme temperature conditions are more frequently encountered, such as in South Asia and sub-Saharan Africa. Little is also known about specific cost-effective interventions that can be implemented in vulnerable communities to reduce adverse outcomes. As the threat of global warming looms large, effective interventions are critically necessary to mitigate its effects.

## 1. Introduction

Global surface temperature has been on the rise since the Industrial Revolution due to the rapid increase in greenhouse gas emissions. The rising trend is likely to continue, increasingly exposing the world population to extreme climatic conditions, more frequently for longer duration and with greater intensity. The 2019 Global Burden of Disease study estimated the burden of extreme temperature, not including the effect of adverse pregnancy or perinatal outcomes because of the lack of causal evidence (1). This manuscript begins with a narrative presentation of the association between temperature and specific pregnancy or perinatal outcomes. In the following section, we discuss the methodological challenges in these types of studies. The final section highlights the gaps in the existing literature. Addressing these gaps should shed light on the nature of the relationship between temperature and adverse outcomes. This manuscript focused on highlighting the gaps and methodology issues in the current literature, rather than conducting a systematic or scoping review, because several of those already exist (2–4).

We focused on the outcomes that were most studied and searched for relevant literature using PubMed, Web of Science, published reviews, and reference lists. We used the following Medical Subject Headings (MeSH): “Temperature,” “Heat,” “Perinatal Outcomes,” “Pregnancy Outcomes,” “Preterm Birth,” “Low Birth Weight,” “Stillbirth,” “Hypertensive Disorders of Pregnancy.” The MeSH terms were combined with “and,” “or,” and “not” operators, and the search was restricted to articles on humans and published before 30 January 2023.

## 2. Preterm birth

We found 24 studies that examined short-term exposure to temperature and preterm birth (PTB) (5–28). The associations reported in these studies are quantitatively different [i.e., hazard ratio (HR), relative risk (RR), or odds ratio (OR)], vary in magnitude and precision, comparison groups are heterogeneous, and exposure ranges from a few individual days before delivery to cumulative average over a period. Eight studies were from the US, seven were conducted on different European populations, three each were from China and Australia, two were from Iran, and one was from Canada (Table 1). The studies vary in size ranging from a few thousand to over a million births, with percentages of PTB between 3% and 17% (Table 1). While many studies included births throughout the year, some included from one season. Four studies from California (9, 16–18) one from Minnesota (14), North Carolina (15), Italy (13), and Spain (6) examined the association separately by season. Most of the studies used birth record data employing retrospective cohort, case-crossover, or time series designs. Individual lag days (e.g., lag 1 representing the day before birth) or cumulative lags (e.g., lag 0–6 representing an average of 7 days, starting from the day of birth and including 6 preceding days), in the weeks and months prior to the day of birth, were used as exposure windows at risk. Additionally, various exposure contrasts were examined, e.g., for heat, 75th percentile or higher, and for cold, 10th percentile or lower temperatures were compared with the percentiles in between (e.g., median). Studies that examined heat waves defined in various ways, e.g., number of days above a threshold, and consecutive number of days above a threshold. The reported associations across studies broadly ranged from 1.01 (1.01, 1.02) to 2.5 (1.02, 6.15) (15, 24); the smallest association was reported by a US study for a 1-day lag temperature of 73–74°C compared with 71–72°C, and the largest was reported by a Swedish study for a 30-day cumulative lag at the 75th percentile (17.5°C) compared with the median (7.7°C). The study-specific associations are presented in Table 1. Notably, most of the studies reported that exposure–outcome associations were below 20%, i.e., the increased risk of PTB was below 20%. While heterogeneity between studies contributed to the diversity of evidence, summarizing results across studies becomes challenging without making additional assumptions. Many exposure windows were often combined with several exposure contrasts, and numerous tests of significance were conducted.

**Table 1:** Summary of studies conducted between 1982 and 2017 on short- and long-term exposure to temperature and preterm or early term births.

**References**	**Location**	**Period**	**Design**	**Total participants, % cases**	**Temperature range**	**Exposure metric/definitions**	**Estimate (95% CI)^#^**
***Short-term exposure studies***							
He et al. (5)	Guangzhou, China	2001–2011	Birth record-based cohort	838,146 singleton vaginal births, 5.6% PTB	1st %tile = 7.6°C; median = 24.4; 99th %tile = 31.9 in °C	1st, 5th, 95th, and 99th %tile compared to the 50th %tile	HR: 17.9% (10.2, 26.2) for 99th; 10.0% (2.9, 17.6) for 1st
Vicedo-Cabrera et al. (6)	Spain	2006–2010 (warm season)	Birth record-based cohort	20,148 singletons; 5.3% PTB	Medians (°C) of the daily min AT^**#**^ = 18.6; max AT = 28.5; mean = 23.6. Lags 0–21 before birth were examined.	50th, 90th, and 99th %tile warm season temp vs. 50th %tile of the complete annual series	RR: 1.20 for Max AT^#^ exceeding 90th %tile for lag 2; 1.05 for min temp exceeding 90th %tile lag 4–6
Mohammadi et al. (7)	Sabzevar, Iran	2011–2017	Birth record-based cohort	3140 PTB, total not reported	Mid study average of daily min (°C) = 12.0; max = 25.0; mean = 25.0. Lags 0–14 before birth were examined.	1st, 25th, 75th and 99th %tile compared to the median of the daily means (19.9°C)	RR range: 1.12 to 1.66 for 99th %tile; 1.14 to 1.79 for 1st %tile, for lags 0 to 3. Other lags of mean temp were not significant.
Huang et al. (8)	50 US metropolitan areas	1982–1988	Matched case-control	1:1 matched pair; 615,329 PTB; 1,005,576 early-term^*^	Min, Mean and Max of daily temp and AT^#^ for the week before delivery, total 60 different exposure combinations	Three HW^#^ definitions. HW1:total hot days; HW2:total consecutive hot days; HW3:7-d average of daily mean minus 97.5 %tile	PTB-broadly no association for HW1–3; Early term-HW1:1.03 (1.01, 1.04) for ≥ 3 days vs. 0 days; HW2: similar as HW1; HW3: 1.05 (1.02, 1.07) per 1°C
Basu et al. (9)	Northern California, US	1995–2009	Time stratified case crossover	14,466 preterm deliveries	Average AT^#^ warm season (°F): 64.5 (5th %tile = 54.2, 95th = 77.1); cold season: 49.2 (5th %tile = 39.7, 95th = 58.4).	Per 5.6°C of cumulative weekly average AT^**#**^. Lag 6 reported.	OR: 1.12 (1.04, 1.20) for warm season and 1.06 (0.98, 1.16) for cold season
Wang et al. (10)	Guangzhou, China	Jan 2015-Jul 2017 (warm season)	Retrospective cohort	215,059 singletons; 1.6% early and 3.6% late PTB^!^	55th %tile (33), 75th (34.6), 90th (35.7), 95th (36.4) and 98th (37) in °C	HW^**#**^: HW1 mean daily max ≥ 33°C; HW2 75th, 95th or 98th %tile ≥ 33°C for 2, 3, 4 consecutive days	HR range from 1.10 (1.01, 1.20) to 1.92 (1.39, 2.64), increasing with intensity of HW
Mathew et al. (11)	Central Australia	1986–2013	Retrospective cohort	16,870 singletons, 8.3% PTB	Median summer temp (°C) = 37; 90th = 40.9, 95th = 41.8, 99th = 43.2. 21 days before birth were examined	Median temp of 30°C was used as the reference temp	RR: 1.08 (1.03, 1.15) for max temp >40°C and 1.37 (1.02, 1.82) for min temp (-6°C) cumulatively for 21 days
Khodadadi et al. (12)	Ahvaz, Iran	2008–2018	Birth record-based cohort	150,766 pregnant women, 5,776 preterm labor	UTCI^#^ range: −40 to 46°C. Lags 0 to 21	25th %tile (19.8°C)	RR: 1.42 (1.01, 2.01) for 1st %tile for lag 0–13. Only one lag of several in the cold season is significant. No association for the hot season.
Schifano et al. (13)	Rome	2001–2010	Birth record-based cohort	132,691 singletons, 5.5% PTB (22nd−36th)	IQR^#^ for daily max AT^#^ for the warm season (°C): 20.4–30.6; and daily min temp for the cold season 3.0–8.8. Lag 0–30 were examined	per 1°C increase in max AT; exposure to HW vs. no HW^@^	% change in the daily PTB counts 1.9% (0.9, 2.9) for lag 0–2; 19.2% (7.9, 31.7) average increase for HW days
Smith et al. (14)	Minnesota	2009–2015	Retrospective cohort	154,157 livebirths, 9.6% PTB	98.6% of women < 37 weeks experienced HI < 37°C; 1.6% of women < 37 weeks experienced HI ≥ 37	Exposure to HI >37°C cumulatively over 7 days prior to delivery	RR: 1.13 (0.99, 1.28)
Ward et al. (15)	North Carolina	2011–2015	Case crossover	256,976 births, 11.6% PTB	Mean temp:82–85°F	Risk at different temp bins on day of birth vs. same day 1 and 2 weeks prior	1% increase at 72–75°F and 1–6% increase in risk at 75–76°F
Basu et al. (16)	California	1999–2006 (warm season)	Case crossover analysis	58,681 PTB	Mean AT^#^ (°F) = 70.4; Max = 88.7, Min = 57.6	Lag 6 exposure, and 3 cumulative average lags	13.5% (10.2, 16.9) risk at 34–36 gestational weeks
Avalos et al. (17)	California	1995–2009	Case crossover	14,466 PTB	Warm season AT^#^ (°F): mean = 64.5, min = 53.4, max = 77.5; Cold: mean = 49.2, min = 40.7, max = 58.5	Mean, max and min AT^#^ for 6-day lag	11.6% (4.1, 19.7) increase for lag06 in warm; 6.2% (−3.0, 16.2) increase in cold
Ilango et al. (18)	California	2005–2013	Birth record-based cohort	1,967,300 mothers with singleton births, 7% PTB	Median (°F) = 83, Min = 43.95, Max = 116.98, IQR = 14.58	12 definitions of HW^#^ at 75th, 90th, 95th, 98th for at least 2, 3, or 4 consecutive days	HR: ranged from 1.008 (1.00, 1.02) at 75th to 1.13 (1.05, 1.21) at 98th for 4 days
Strand et al. (19)	Brisbane, Australia	2005–2009	Birth record-based cohort	101,870 births, outcome PTB	Mean (°C) = 21; Min = 15.4; Max = 25.4	PTB at 27°C and 16°C vs. 21°C	HR: 1.20 at 27°C, 0.97 at 16°C
Wolf et al. (20)	Germany (Brandenburg and Saxony)	2002–2010	Time-series analysis	PTB: B'burg (128,604, 6.8%); Saxony (162,913, 6.3%)	Mean (°C): Brandenburg = 9.1–9.6; Saxony = 9.0–9.7	Quintiles 2, 3, 4, 5 of mean temp vs. 1st for 1st month, 1st trim, and linear per °C	OR: week before birth 1.00 (0.93, 1.08), similar ORs for 1st month, 1st trim exposures
Wang et al. (21)	Brisbane, Australia	2000–2010	Population based ecological	50,848 births, 16.9% PTB	Average weekly max temps (°C): Min = 14, Q1 = 23.1, Q2 = 26.1, Q3 = 28.5, Max = 37.9	9 different HW definitions	HR: ranged from 1.13 (1.03, 1.24) to 2.00 (1.37, 2.91) for different HW definitions
Auger et al. (22)	Montreal, Canada	1981–2010 (warm season)	Retrospective cohort (time-to-event)	206,929 term and 12,390 PTB singletons	Max daily temp (°C): Median = 27.7, IQR = 4.4, Min = 10.1, Max = 35.4	3 consecutive days ≥ 32°C in the week before birth (yes vs. no)	HR: Early term: 1.17 (1.06, 1.29); PTB 0.92 (0.74, 1.14). Higher HRs for 4–7 such days in that week
Kloog et al. (23)	Massachusetts	2000–2008	Birth record-based	473,977 births (~4.3% PTB)	Mean (°C) = 11.3, Median = 11.4, Min = −12.1, Max = 35.49, 25th = 6.9, 75th = 1.6	Closest monitor temp for entire pregnancy, per 2.7°C	OR: 1.02 (1.00, 1.05) per 2.7°C increase in monitor-based temp
Vicedo-Cabrera et al. (24)	Stockholm, Sweden	1998–2006	Birth record-based	95,069 birth, 3.5% PTB	Min (°C) = −21.5, 25th = 1.5, Median = 7.7, 75th = 14.5, Max = 25.6	Temp 75th %tile vs. median (7.7°C); 30-day lags were examined	RR cumulative RR: 2.5 (1.02, 6.15)
Arroyo et al. (25)	Madrid, Spain	2001–2009	Time series	298,705 births; 8.2% PTBs	Min (°C) = −6.1, max = 38.6; HW->34, CW- < -2	HW daily max temp >34°C vs. ≤ 34	RR: 1.055 (1.018, 1.092) for lag 1 when it was >34°C
Cox et al. (26)	Flanders, Belgium	1998–2011	Birth record-based	807,835 live births, 5.8% PTB	Min temp (°C) = −12.3 to 23.9, Median 8.3, Max temp = −6 to 35.8 with median 14.7	95th, 99th vs. median 14.7°C; 5th, 1st vs. 8.3; cumulative effects over lag 0–1, 0–3 and 0–6 days	RR: 1.09 (1.01, 1.15) for 95th and 1.19 (1.05, 1.36) for 99th for lag 0–6; No effect for cold.
Liang et al. (27)	Shenzhen, China	2005–2011	Time series	1,040,638 singletons, 5.6% PTB	Min (°C) = 5.4, Median = 24.5, Max = 32.7	Lag days: 0, 5, 10, 15, 20, 25, and 30. 1st, 5th, 95th, 99th %tiles vs. 24.5°C (median as ref)	RR: 1.72 (1.28, 2.33) for 1st vs. median; 0.62 (0.52, 0.75) for 99th vs. median for lag 0–30 days
Schifano et al. (28)	Rome, Barcelona, Spain	2001–2012	Population-based survival analysis	Rome: 78,633 (5.5% PTB); Barcelona: 27,255 (4.5% PTB)	Barcelona (°C): Max = 37.5, Min = 3.5, Median = 25; Rome: Max = 39.5, Min = 4, Median = 25.5	HRs for PTBs for 1°C increase in max AT	HR: 1.03 (1.02, 1.04) for Barcelona and 1.03 (1.02, 1.05) for Rome for lag 0–2
***Long-term exposure studies***							
He et al. (5)	Guangzhou, China	2001–2011	Birth record-based cohort	838,146 singleton vaginal births, 5.6% PTB	1st %tile = 7.6°C; median = 24.4; 99th %tile = 31.9 in °C	1st, 5th, 95th, and 99th %tile compared to the 50th %tile	HR: 1.10 (1.02, 1.18) for 99th; 1.12 (1.05, 1.21) for 1st
Kwag et al. (29)	Korea	2010–2016	Retrospective cohort	1,329,991 births, 4.6% PTB	HW lasted more than 2 days with a max temp ≥ 33°C per day	Exposure to HW^#^ hours into 4 categories: <70 %tile (ref), 71–80, 81–90, >90; <67 %tile (ref), 68–78, 79–88, >88	OR: 1st trim−1.05 (1.01, 1.19) for 81–90th %tile and 1.05 (1.01, 1.09) for >90th; 2nd trim−1.04 (1.00, 1.07) for 79–88th and 1.17 (1.13,1.22) for >88th
Spolter et al. (30)	Israel	May 2004-Mar 2013	Birth record-based cohort	62,547 singletons; 0.8% early PTB, 6.9% late PTB, 25% early term^&^	Mean daily temp (°C) avg over entire pregnancy range 12.6–29.1, mean 19.8	Temp quintiles, middle quintile as ref	HR: 1.31 (1.11, 1.56), 1.13 (0.98, 1.29) for late PTB for 5th and 4th 1.24 (1.13, 1.36) and 1.16 (1.07, 1.25) for early term
Cushing et al. (31)	Texas, US	2007–2011	Retrospective cohort	198,013 singletons, 10.3% PTB	99th %tile for daily min (°C) temp – 25.5, max – 37.2 for summer	HRs for risk of PTB for max AT>40°C compared to < 20	HR: 1.15 (1.01, 1.30). Lower temps were not associated.

^*^Early PTB was defined as 23–30 weeks; late PTB as 31–36 weeks; and early term as 37–38 weeks. ^#^CI, confidence interval; HW, heat wave; HI, heat index; AT, apparent temperature; UTCI, universal thermal climate index; IQR, interquartile range, CW, cold wave. ^!^Early PTB between weeks 28 and 34, and late PTB between weeks 35 and 37. ^@^HWs were identified as at least two consecutive days with max AT above the monthly 90th percentile or the daily min temp above the monthly 90th percentile and max AT above the median monthly value of the available series (1987–2010, excluding 2003). ^&^Early preterm (23–30 + 6/7 weeks), late preterm (31 + 0/7–36 + 6/7 weeks), and early term (37 + 0/7–38 + 6/7, weeks).

Four studies examined the association between long-term exposure to temperature and PTB (Table 1) (5, 29–31). Associations ranging from 1.05 (1.01, 1.19) to 1.31 (1.11, 1.56) were reported in studies conducted among Chinese, Korean, Israeli, and US populations (5, 29–31).

Effect modification by infant sex, race, ethnicity, maternal characteristics, and air pollution exposure has also been reported (8, 9, 26, 31). Three studies reported the associations with temperature were higher for non-Hispanic African Americans and Hispanics compared with non-Hispanic white people (8, 9, 31). Maternal smoking, alcohol consumption, hypertension, and diabetes have also been suggested to increase susceptibility to temperature (9). Preliminary evidence of synergism between heat waves and PM_2.5_ has been reported (10, 29). However, in another study, neither PM_10_ nor ozone modified the association between apparent temperature (AT) and PTB, although maternal age, education, and pre-existing chronic diseases modified the association (13).

## 3. Birth weight, low birth weight, and small for gestational age

We found 16 studies that examined the association of temperature with birth weight or low birth weight (LBW) (Table 2). Four of these studies were from the US, four from Europe, two from Israel, two from South America, two included global populations, and one each from Australia and Bangladesh (20, 23, 32–45). The majority of these studies included births occurring within the last two decades. However, the two global studies used World Health Organization (WHO) datasets and included births between 1980 and 1992 (41, 42). Most of the studies used retrospective cohort designs, except two, which were time-series (20) and prospective (37). Some studies reported associations of entire pregnancy exposures with LBW or birth weight (23, 35, 36, 38), while others reported associations with trimester-specific exposures (20, 23, 32–44). The associations for LBW broadly ranged between 18% (−5, 45%), per 2.4°C increase, and 148% (119, 181%) for temperature below the 5th percentile compared with 5th and 95th percentiles (Table 2) (35, 43). The study-specific associations along with the exposure contrasts are presented in Table 1. Notably, some studies included all LBW infants, regardless of gestational age, while others used term LBW (born at or after 37 completed weeks).

**Table 2:** Summary of studies conducted between 1985 and 2018 examining the association between temperature and birth weight, low birth weight or small-for-gestational age.

**References**	**Location**	**Period**	**Design**	**Total participants, % cases**	**Temperature range**	**Exposure metric/definitions**	**Estimate (95% CI)^#^**
***Birth weight and low birth weight***							
Basagaña et al. (32)	Israel	2010–2014	Retrospective cohort	624,940 singleton births, 2.7% tLBW	Daily mean temps (°C): median ranged from 15.3–25.3; 10th %tile ranged from 13.2–22.5; 90th ranged from 18.0–28.5	1st and 10th deciles compared to 5th	OR: 1.35 (1.22, 1.49) for 1st; 1.58 (1.42, 1.74) for 10th
Kloog et al. (33)	Israel	2004–2013	Birth record-based	56,141 singleton term births, 3.1% tLBW	Mean daily temp (°C): Median = 19.9; Max = 24.9; Min = 14.6	Lowest (<18.5°C) and highest quartile (>21.3°C) vs. Q2 and Q3	OR: 1.33 (1.11, 1.58) for lowest and 1.17 (0.99, 1.38) for highest
Bakhtsiyarava et al. (34)	Latin American cities	2010–2015	Birth record-based	Brazil-8,079,872; Mexico- 6,405,777; Chile-890,156	Average monthly (°C) during gestation: Brazil = 22.2, Mexico = 18.9, Chile = 14.0	Difference in birth weight at a country specific 5th, 50th, 95th %tile compared to 19°C	tBW reduction: Brazil: 10 g at 50th, 37 g at 95th; Mexico: 3 g at 50th, 9 g at 95th; Chile: 26 g at 50th %tile
Ha et al. (35)	12 US sites	2002–2008	Retrospective cohort	220,572 singleton births, 2.2% tLBW	Avg daily temp (°C): mean = 13.3, min = −2.3, max = 28.2, 5th %tile = 5.2, 95th = 24.7, IQR = 8.0	For each site and time window, cold (< 5th %tile), hot (>95th %tile), compared with mild (5–95th)	RR: Cold: 148% increased risk; Hot 138% increased risk
Sun et al. (36)	USA	1989–2002	Retrospective cohort	29,597,735 singleton births	Overall average temps during entire pregnancy (°F): min = 46.1, median = 55.5, max = 64.9	Deciles: cold (< 20th %tile), hot (>80th %tile), relative to 40th to 50th %tile for that county	tBW reduction: 15 g for >90th and 6 g for < 10th %tile
Li et al. (37)	Australia	2001–2010	Prospective	237,585 pregnant women	Min = 4.7°C; Max = 33.4°C	Exposure in the 1st gestational week vs. first 4 weeks and last 1 week vs. last 4 weeks	BW: 20–25°C and <20°C increased BW by 0.011 kg and 0.018 kg, respectively, compared to >30°C
Ngo et al. (38)	New York city	1985–2010	Birth record-based	>500,000 births	Cold: < 25°F; Hot:>85°F	Exposure to an extra day where average < 25°F or >85°F relative to more comfortable temperatures (45–65°F)	BW reduction: 0.8 g at < 25°F in trim 1 and 1.8 g cumulative effect; 1.1 g at >85°F in trims 1 and 2 and 1.7 g as cumulative effect
Arroyo et al. (39)	Madrid	2001–2009	Time-series analysis	298,705 births, 13.3% LBW	Max daily temp = 36.7°C, Min daily temp = −2°C	Per 10°C increase in max temp	RR: 1.003 (1.002, 1.004) for 2nd trim
Rashid et al. (40)	Bangladesh	2001–2004	Prospective cohort	3267 singleton, 30.5% LBWs	Mean (°C): winter = 22.4, hot = 27.4, monsoon = 28.5	per 1°C increase in mean daily temperature	5 g increased BW at week 19; 0.04 cm decreased and 0.05cm increased BL^#^ at week 8 and at birth, respectively
Wells and Cole (41)	Global	1992 WHO data	Unclear design	140 diff populations, median births = 5558	Mean (SD) HI = 1.51 (1.18);	Mean humidity adjusted temp per year for all locations	Correlation = −0.59 (*p* < 0.001); 9.6% between-population variance
Wolf and Armstrong (20)	Germany (Brandenburg and Saxony)	2002–2010	Time-series analysis	B'burg (128,604); Saxony (162,913); 4.9% LBW both cities	Mean (°C): Brandenburg = 9.1–9.6; Saxony = 9.0 to 9.7	Quintiles 2, 3, 4, 5 of mean temp vs. 1st for 1st month, 1st trim, and per °C	OR: trim1 0.93 (0.70, 1.23), similar ORs for other trimesters
Jensen and Sørensen (42)	Global	1992 WHO data	Birth record-based	192 countries, 125 included BW	Range (°C) of Min: −25 to 25 and Max: 13 to 47	per °C increase in min and max temp	BW reduction: 6 g and 23 g per 1°C increase in min and max temp, respectively
Dadvand et al. (43)	Spain	2001–2005	Birth record-based	6438 singletons; 3% tLBW	Median (°C): Ranged from 33.1 to 35.6	per 2.4°C IQR increase	OR: 1.18 (0.95, 1.45)
Kloog et al. (23)	Massachusetts	2000–2008	Birth record-based	453,658 term births	Mean (°C) = 11.3, Median = 11.4, Min = −12.1, Max = 35.49, 25th = 6.9, 75th = 1.6	Entire pregnancy : closest monitor temp per 2.7°C, modeled temp per 8.4°C	16.7 g lower BW per 8.4°C in 3rd trim modeled temp; OR for LBW: 1.04 (0.96, 1.13) per 2.7°C in model-based temp exposure over entire pregnancy
Molina and Saldarriaga (44)	Andean region, South America	1990–2013	Birth record-based	86,021 live births, 7% LBW	Avg temp 9 months before birth (°C), Mean = 19.4, Range = −4.9 to 31.5	Effect of temp variability^@^ on BW and LBW probability at different periods during pregnancy	BW reduced by 19.7 g (entire preg), 16.5 g (1st trim), 10.3 g (2nd trim); LBW probability: 0.07% (entire preg), 0.03% (1st trim), 0.05% (2nd trim)
Poeran et al. (45)	Netherlands	2000–2008	Retrospective cohort	1,460,401 births	Max daily (°C) = 15.9 to 37.8, Min daily = −20.7 to 4.4	Reference group was not clearly mentioned	BW reduction: Min temp −0.6 (−1.1, −0.2) in 1st trim, 0.6 (0.3, 1.0) in 2nd trim, 1.5 (1.3, 1.8) in 3rd trim; Max temp −1.6 (−1.9, −1.2) in 1st, −2.2 (−2.5, −1.9) in 2nd, −2.0 (−2.3, −1.7) in 3rd trim
***Small for gestational Age***							
Kloog et al. (33)	Israel	2004–2013	Birth record-based	56,141 singleton term births, 15.4% SGA	Daily mean temp: Median (°C) = 19.9; Max = 24.9; Min = 14.6	Lowest (< 18.5°C) and highest quartile (>21.3°C) compared to Q2 and Q3	OR:1.18 (1.09, 1.29) for < 18.5°C; 0.91 (0.84, 0.99) for >21.3°C
Ha et al. (46)	12 US sites	2002–2008	Retrospective cohort	220,572 singleton births, 11.2% SGA	Avg daily temp (°C): mean = 13.3, min = −2.3, max = 28.2, 5th %tile = 5.2, 95th = 24.7, IQR = 8.0	For each site and time window, cold (< 5th %tile), hot (>95th %tile), vs. mild (5–95th %tile)	No clear association for hot exposure; OR: 0.91 (0.86, 0.97) for cold exposure in 3rd trim
Sun et al. (36)	US	1989–2002	Retrospective observational	29,597,735 singleton births, 10.2% SGA	Overall average temps during entire pregnancy (°F): min = 46.1, median = 55.5, max = 64.9	Cold (< 20th %tile), warm (>80th %tile) relative to decile spanning 40th to 50th %tile	OR:1.03 (1.02, 1.04) for 80th-90th; 1.04 (1.03, 1.05) for >90th; 0.995 (0.985, 1.004) for 10th-20th and 1.004 (0.992, 1.016) at < 10th %tile
Chen et al. (47)	China	2014–2018	Multi-center prospective cohort	179,761 mother-infant pairs, 10.4% SGA	Mean (°C) during whole pregnancy = 17.62, 25th = 14.86, 50th = 18.40, 75th = 20.61	Exposure for Heat:>90th and 95th %tile; Cold- < 10th and < 5th %tile vs. 41st −50th %tile	OR: Highest of 1.03 (1.01, 1.05) during 21st and 22nd weeks for heat; inverse for cold during 1st-8th weeks with lowest 0.963 (0.934, 0.994) during 1st
Dadvand et al. (43)	Spain	2001–2005	Birth record-based	6438 singletons; 12% SGA	Median (°C): Ranged from 33.1 to 35.6	per 2.4°C IQR increase	OR: 1.08 (0.96, 1.20) for SGA
Molina and Saldarriaga (44)	Andean region, South America	1990–2013	Birth record-based	86,021 live births, 6% small at birth	Avg temp 9 months before birth (°C), Mean = 19.4, Range = −4.9 to 31.5	Probability of small at birth at different exposure periods	Probability: 0.09% (whole pregnancy), 0.03% (1st trim), 0.06% (2nd trim)

^*^LBW was defined as BW < 2500 g and SGA as BW < 10th percentile for gestational age. tLBW refers to term low birth weight. ^#^CI, confidence interval; HW, heat wave; AT, apparent temperature; HI, heat index; BL, Birth Length. ^@^Temperature variability defined as the number of standard deviations relative to the municipality's historical mean (average temperature for the period 1950–2010).

Two studies examined short-term exposure (Table 2). An Australian study reported the association between a daily maximum temperature of 20–25°C in the last week of pregnancy and 11 g (8, 18) higher estimated mean birth weight compared with > 30°C (37); another study from Spain reported an RR of 1.003 (1.002, 1.004) for LBW per 10°C increase in maximum temperature on the 14th week (39).

We found six studies from the US, Europe, Israel, China, and South America that investigated small for gestational age (SGA) (Table 2) (33, 35, 36, 43, 44, 47) with ORs ranging from 1.03 (1.01, 1.05) to 1.18 (1.09, 1.29).

## 4. Spontaneous abortion and stillbirth

We found 13 studies that examined the association of temperature with spontaneous abortions and stillbirths (12, 39, 46, 48–57) (Table 3). A unique study from Hungary reported that the likelihood of unobserved pregnancy loss is 0.22 (0.12, 0.33) per 100, 000 women aged 16–44 years if there is 1 day in the first 2 weeks of pregnancy with a mean temperature of >25°C compared with 15–20°C (48). Compared with the other outcomes, relatively more studies on stillbirth are from low- and middle-income countries, likely because of the higher incidence of stillbirth in these regions. These studies employed cross-sectional, case-crossover, retrospective, or time-series designs (Table 3). The associations for stillbirth ranged from 1.03 (1.00, 1.06), when the temperature was above 97.5th percentile for 4 consecutive days in the week prior to birth compared to 0 days above that level, to 3.7 (3.1, 4.5), per 1°C increase in the mean temperature in the week prior to birth in the warm season (46, 55).

**Table 3:** Summary of studies conducted between 1981 and 2021 examining the association between temperature and stillbirth.

**References**	**Location**	**Period**	**Design**	**Total participants, % cases**	**Temperature range**	**Exposure metric/definitions**	**Estimate (95% CI)^#^**
Hajdu and Hajdu (48)	Hungary	1981–2015	Birth record-based	6,554,519 pregnancies, 9% pregnancy loss	8 temp categories (°C); lowest = <5, highest = >25	Exposure in the 1st 2 weeks to an additional hot day (>25°C) vs. all days with mean 15–20°C	0.22 (0.12, 0.33) per 100,000 when one hot day in 1st 2 weeks of pregnancy
Asamoah et al. (49)	Ghana	2004–2007	Cross-sectional	1,136 pregnancies, 9.6% miscarriage; 2.8% stillbirth	Mean yearly WBGT (°C) = 26.1–27.5; Max yearly WBGT = 24–26.1	per 1°C degree increase in WBGT	OR: 1.12 (0.90–1.39)
Nyadanu et al. (50)	Ghana	2020–2021	Time series	5,961,328 births, 1.51% stillbirth	Mean UTCI^#^ (°C) = 28.5, Median = 28.8	Exposure to 1st−25th %tile UTCI^#^ and 99th %tile compared to median	2 to 18% risk at 90th %tile
Karlsson et al. (51)	Northern Sweden	1880–1950	Historical case-crossover	141,880 births, 2.27% stillbirth	Min daily temperature (°C) = −31.8°C, Max daily temperature = 25.1	IRR^#^ over a temperature range relative to minimum mortality temp (+15°C)	IRR: 2.3 (1.28, 4.00) at −10°C from April to September
Rammah et al. (52)	Texas	2008–2013	Case-crossover	709 stillbirths, 12.27% placental abruption	Mean daily AT (°F): Max = 101.1; Min = 48.7	AT^#^ exposure on each of lag days 1 to 6	OR: 1.37 (1.16, 1.61) and 1.25 (1.07, 1.47) for lags 1 and 4, respectively per 10°F
Auger et al. (53)	Quebec, Canada	1981–2011	Case-crossover	5,047 stillbirths	Max temp on the day before death (°C) = −12.4 to 36.1	Max temp on the day before death relative to 20°C	OR: 1.16 (1.02, 1.33), 1.22 (1.02, 1.46), and 1.28 (1.03, 1.60) at 28°C, 30°C, and 32°C, respectively
Ranjbaran et al. (54)	Iran	2015–2018	Time series	516,570 births, 0.67% stillbirths	Daily temp (°C): Min = −7.22, Max = 42.22, Mean = 17.7	RR at 99th, 95th, 75th, 25th, 5th, and 1st %tiles relative to corresponding median	RR: highest of 1.25 (0.95, 1.65) at 99th with median acc to min daily temp; 0.92 (0.72, 1.19) at 1st with median acc to max daily temp
Khodadadi et al. (12)	Ahvaz, Iran	2008–2018	Birth record-based	150,766 pregnancies, 1.30% stillbirth	Mean UTIC (°C) = 29, Median = 28.6, Min = 5.8, Max = 49.8	Effect of 99th relative to 75th (38.0°C) and effect of 1st relative to 25th (19.8°C)	RR: 2.05 (1.01, 4.15) for thermal stress (99th vs. 75th)
Richards et al. (55)	6 US states	1991–2017	Matched case-control	140,428 stillbirths	Mean (°C): CA = 14.5, FL = 21.6, GA = 17.8, KS = 12.8, NJ = 11.9, OR = 9.5; Hot day if mean temp above county-specific 97.5th %tile	HW1 (exposure for 1, 2, >3 vs. 0 days), HW2 (>2, >3 or >4 consecutive days vs. no exposure), HW3 (continuous exposure per 1°C increase above threshold)	OR: 1.03 (1.00, 1.06) for 4 consecutive days in the week prior to birth, which increases with intensity and duration
Ha et al. (35)	12 US cities	2002–2008	Multicenter retrospective	223,375 singletons, 0.44% stillbirths	Cold (< 10th %tile), hot (>90th %tile), and mild (10th−90th %tile)	Chronic exposure: hot and cold vs. mild; Acute exposure: mean temp week prior to delivery	OR: 3.71 (3.07, 4.47) and 4.75 (3.95, 5.71) for hot and cold respectively; 1.06 (1.03, 1.09) per 1°C increase in mean temp in the week prior to birth in warm season
Arroyo et al. (39)	Madrid	2001–2009	Time series	298,705 births, 0.41% late fetal death	Max daily temperature (°C) = 36.7; Min daily temperature = −2	per 10°C in max temp	RR: 1.037 (1.035, 1.039) and 1.012 (1.010, 1.013) for min and max temp in 3rd trim
Savitz and Hu (56)	Florida	2012–2017	Case-crossover	1,876 stillbirths	Max HI in August = 31.7°C	Average HI in lag day 1 through 7	OR 1.17 (1.05, 1.31) per 1°C increase in HI in summer for socioeconomically deprived
Strand and Barnett (57)	Brisbane, Australia	2005–2009	Birth record-based	101,807 births, 0.6% stillbirth	Mean monthly temp: Mean (°C) = 21; Max = 25.4; Min = 15.4	HR for stillbirth at < 28 weeks, 28–36 weeks and >37 weeks vs. 21°C	HR: 1.20 at 27°C during 28–36 weeks; 0.97 at 16°C during >37 weeks

^*^Stillbirth was defined as fetal death at or after 20 weeks of pregnancy. ^#^CI, confidence interval; IRR, incidence risk ratio; AT, apparent temperature; HW, heat wave; LFD, late fetal death; HI, heat index.

The association between temperature and stillbirth has been reported to differ by race, ethnicity, or socioeconomic status. Higher associations were reported for non-Hispanic African Americans and Hispanics compared with no association for non-Hispanic white (52). Higher association was also reported only in most socioeconomically deprived population but not in the overall study population (56).

## 5. Hypertensive disorders of pregnancy

Temperature has been associated with hypertensive disorders of pregnancy. Two- to three-fold increase in the risk of preeclampsia per 9°C has been found for 1st, 2nd, and 3rd trimester averages of daily mean temperatures (58). Before 12 weeks of conception, colder temperature has been reported to increase the odds of preeclampsia or eclampsia (59). However, in the first 20 weeks of pregnancy, colder temperature reduced the odds, while hotter temperature increased the odds of preeclampsia or eclampsia (59). No association with preeclampsia was found for heat stress in the 3 weeks prior to delivery (12).

## 6. Methodological considerations

Choice of metric is an important consideration because each represents qualitatively different exposure. For example, AT incorporates humidity and reflects how hot it feels outdoors. High humidity in moderately warm conditions can cause discomfort leading to physiological stress (60). High minimum temperature over consecutive days reflects the lack of overnight cooling, which could cause sleep perturbations (61). High maximum outdoor temperature increases physical discomfort, but wind velocity may increase or decrease physiological stress. When the body is sweating, wind can considerably influence regulatory physiological mechanisms. The other less commonly used metrics to measure heat stress are excessive heat factor, physiological equivalent temperature (PET), and Universal Thermal Climate Index (UTCI) (12, 62, 63). Regardless of the primary choice of metric, at least a few (e.g., maximum and minimum AT) should be uniformly reported to allow comparisons of association across studies. Furthermore, if temperature thresholds or non-linear relationships are observed, additional results should be reported in a manner that will allow between study comparisons and pooling of estimates across studies.

The importance of the comparison group can be highlighted using the example of the tri-country Latin American study (34). In the study, the means of the monthly averages for Brazil, Mexico, and Chile were 22, 19, and 14°C, respectively. Because of the annual weather patterns in each country, for Brazil, the comparison between the 50th percentile (22) and the reference group (19°C, average across all study sites) reflects the effect of heat, while for Chile, the same comparison (14) reflects the effect of cold. Thus, based on the same exposure contrasts, the results are essentially different effects of temperature.

Measuring exposure accurately is an enduring challenge in environmental epidemiology. Most studies use fixed site or satellite measurements as proxies for true personal exposure (30, 33, 58). Small deviations (~0.5°C) in true exposure may have significant repercussions on physiological processes during critical windows of pregnancy, which, if missed, can produce misleading results. Residential mobility during pregnancy, which has been studied more in the context of air pollution, may also affect exposure to extreme temperature (64–66). The presence and extent of exposure misclassification depend on the proportion-changing residences during pregnancy, which vary by region (67, 68). The amount of time spent in temperature-controlled environments could also misclassify exposure, which requires prospective studies designed to measure the time activity patterns of the participants. This information is seldom available from routine sources such as birth registries or hospital records.

Studies should use standardized outcome definitions, especially when countries use different definitions, e.g., stillbirths are recorded at 20 (US), 24 (UK), or 28 (WHO) weeks of gestation (46, 49, 51–56). Another example is late fetal death defined in a study as “death within the first 24 h of life” (39), which is inconsistent with the definition by WHO that states the first 24 h after birth as the early neonatal period. The availability of reliable data on spontaneous abortion and stillbirth is a challenge across regions. Accuracy of gestational age determines the classification of PTB, especially when the last menstrual period-based assessment is not verified by first trimester ultrasound. Studies examining temperature and birth weight should conceptually consider gestational age because it has been suggested to be in the causal pathway and adjustment could produce biased estimates (69).

As temperature, air pollution, and season are inter-related, to understand the independent associations, studies should develop frameworks including all known and potential risk factors in the context of the temperature–outcome relationship (e.g., Directed Acyclic Graphs). Temperature and air pollution vary by season. Perinatal outcomes have also been observed to vary by season, as do births (70). Several studies adjusted for air pollution, while others examined effect modification (10, 13, 28, 35, 39, 54). Buckley et al. argued that temperature and some pollutants (e.g., ozone) likely share predecessors (71). The nature of the pollutant warrants careful consideration before inclusion with temperature in the same model. The same argument also applies to seasonality and temperature. Restricting births to a season is an option, though it limits generalizability and sample size.

Fixed cohort bias is a threat to validity, but it has generally been considered (19, 72). It occurs when births, only within a fixed period, is included. This results in shorter pregnancies being missed at study initiation, while longer pregnancies are missed at the end of the study (19). To avoid fixed cohort bias, studies have used conception dates within a fixed period or excluded pregnancies conceived before a fixed time prior to the starting date of the study (5, 8, 55).

## 7. Gaps in current knowledge

From the perspective of exposure to extreme temperatures, several questions remain unanswered. Several studies have reported associations with multiple exposures windows, separately. It remains unclear, which exposure(s) is(are) relatively more harmful - a single extreme, several consecutive extremes, several interspersed extremes or moderately high prolonged exposures? Examining the overlap of these extremes with stages of fetal development will advance the understanding of the pathophysiological pathways. Fetal growth is programmed around the first month, when the placental bed is formed, which could be sensitive to exogenous exposures and merits investigation. Conversely, lean body mass growth occurs in the third trimester, a period that is also vulnerable to environmental exposures. It will be analytically complex to examine more than one exposure simultaneously (e.g., short- and long-term exposures), especially when they are biologically relevant and statistically correlated. Models have been developed that can examine multiple correlated exposures (e.g., Bayesian Kernel Machine Regression and distributed linear and non-linear lag models) (73–75).

Studies have provided some insights into potential biological mechanisms. For example, heat has been shown to affect thermoregulation and blood pressure during pregnancy (46, 76–78). Around 37 weeks when thermoregulation becomes less efficient because of increased body mass and decreased ability to dissipate heat by sweating, heat-related uterine contraction has been observed (28, 79). Extreme temperature exposure during pregnancy has been linked with systemic inflammation, cell impermeability, upregulation of heat shock proteins, release of endotoxins (46, 80), and gene sequencing (81, 82). Nonetheless, we do not know how core body temperature modulates under extreme ambient temperature, which could be an intermediate link between exposure and biological mechanisms. The fetus is usually ~0.5°C hotter than the maternal core and can lose heat only through the umbilical artery and amniotic fluid (83). In the event of maternal core temperature increase, heat likely gets transferred to the fetus (84, 85). Any physiologic or systemic effects interfering with maternal—fetal exchange will likely affect fetal development, growth, and potential survival. We do not know the magnitude of non-modulation of body temperature that is clinically important, i.e., when the body starts triggering physiological counterbalances. Does non-modulation differ in different populations and what factors (e.g., lifestyle characteristics) influence it? None of these questions have been addressed to date. Bonell et al. (86) are taking an important stride in that direction. More studies are needed in different populations to understand the modulation of core body temperature during pregnancy, transfer of excess heat from the mother to the fetus, and its consequences. Heat sensing and pregnancy monitoring wearables have been feasibly implemented in various settings including in low- and middle-income countries (87–92). These wearable devices are small. Prices range from fifty to several hundred US Dollars and come in various forms such as watches, rings, bracelets, and small buttons. The devices can store a reasonable amount of data, and the frequency of measurement determines the duration of a cycle. Technology involved in transferring data from these devices for further use in research varies in complexity. In addition to body temperature, some of these devices can measure ambient temperature, sleep, physical activity, etc. Technology has opened up avenues for accurate measurement of environmental exposures and helped advance research, though it is necessary to test these devices for accuracy before extensive use.

Populations acclimatize to their natural surroundings. A maximum temperature that is heat wave in temperate zones will be perceived differently in tropical countries. Localized behavioral adaptations during extreme heat or cold, such as modifying outdoor activities and consumption of specific food types, help mitigate adverse effects, predisposing populations to varying levels of risks. Most of the existing studies were conducted in the Western Hemisphere. New studies should be conducted on populations exposed to extreme temperatures and in low- and middle-income countries to understand the global risk pattern.

Little is known about the cost-effective interventions that can be implemented in vulnerable communities to reduce adverse outcomes. Vulnerable communities are likely to have fewer mitigation measures (e.g., air conditioning and lack of access to health care), particularly in rural and socioeconomically deprived areas. These communities are also likely to have a higher prevalence of pre-existing conditions and high-risk lifestyle (e.g., substance use and drinking), disposing pregnant women to higher risk, regardless of heat exposure.

For a comprehensive understanding of the nature of the effect, studies should be conducted in places where populations are frequently exposed to extreme temperature. Several other gaps in the available literature exist, including no knowledge about modulation of core body temperature during pregnancy, when exposed to extreme ambient temperature, which can help understand the biological mechanisms. Even if the epidemiologic associations of temperature with PTB, LBW, SGA, and stillbirths are small, these are clinically relevant indicators of newborn health and are observed across populations in varying magnitude. Despite a small association, continuing global warming will potentially increase exposure prevalence, thereby increasing the death and disability burden attributable to temperature, as well as the associated healthcare cost of the affected infants.

## Data availability statement

The original contributions presented in the study are included in the article/supplementary material, further inquiries can be directed to the corresponding author.

## Ethics statement

Ethical approval was not required for the study involving humans in accordance with the local legislation and institutional requirements. Written informed consent to participate in this study was not required from the participants or the participants' legal guardians/next of kin in accordance with the national legislation and the institutional requirements.

## Author contributions

MM involved in literature search, drafted, and revised the manuscript. RB provided expert critique and suggested revision of the manuscript. RG conceptualized the research ideas and revised the manuscript. All authors contributed to the article and approved the submitted version.
